# Case Report: Persisting Lymphopenia During Neuropsychiatric Tumefactive Multiple Sclerosis Rebound Upon Fingolimod Withdrawal

**DOI:** 10.3389/fneur.2021.785180

**Published:** 2021-10-29

**Authors:** Valeria Koska, Moritz Förster, Katja Brouzou, Ercan Arat, Philipp Albrecht, Orhan Aktas, Patrick Küry, Sven G. Meuth, David Kremer

**Affiliations:** Department of Neurology, Medical Faculty, University Hospital Düsseldorf, Heinrich Heine University, Düsseldorf, Germany

**Keywords:** multiple sclerosis, rebound, tumefactive, lymphopenia, neuropsychiatric, fingolimod

## Abstract

Fingolimod (FTY) is a disease modifying therapy for relapsing remitting multiple sclerosis (RRMS) which can lead to severe lymphopenia requiring therapy discontinuation in order to avoid adverse events. However, this can result in severe disease reactivation occasionally presenting with tumefactive demyelinating lesions (TDLs). TDLs, which are thought to originate from a massive re-entry of activated lymphocytes into the central nervous system, are larger than 2 cm in diameter and may feature mass effect, perifocal edema, and gadolinium enhancement. In these cases, it can be challenging to exclude important differential diagnoses for TDLs such as progressive multifocal leukoencephalopathy (PML) or other opportunistic infections. Here, we present the case of a 26-year-old female patient who suffered a massive rebound with TDLs following FTY discontinuation with primarily neuropsychiatric symptoms despite persisting lymphopenia. Two cycles of seven plasmaphereses each were necessary to achieve remission and ocrelizumab was used for long-term stabilization.

## Introduction

Fingolimod (FTY), an effective oral disease modifying therapy (DMT) for relapsing remitting multiple sclerosis (RRMS), sequesters lymphocytes in lymphatic tissue such as the lymph nodes. Accordingly, in some cases it can lead to severe lymphopenia. This prompts many neurologists to discontinue therapy in order to avoid opportunistic infections even though clear proof of such a risk is still lacking (1). Since 2012, several cases have been reported describing severe disease reactivation following FTY withdrawal featuring tumefactive demyelinating lesions (TDLs) (2, 3). TDLs are defined as demyelinating lesions larger than 2 cm in diameter and may feature mass effect, perifocal edema and gadolinium (Gad) enhancement (4). Important differential diagnoses for TDLs are progressive multifocal leukoencephalopathy (PML) and/or opportunistic infections. In almost, all of these cases disease reactivation is accompanied by rapid lymphocyte reconstitution. Here, we present the case of a 26-year-old female patient (after obtaining written and informed consent) who suffered a massive rebound with subcortical edematous TDLs after FTY discontinuation due to lymphopenia which persisted for more than 6 weeks after therapy was halted. Her clinical symptoms were primarily neuropsychiatric including affective incontinence and motoric aphasia. To our knowledge, only one similar case was reported in the literature by Ashtari et al. (3).

## Case Presentation

**Figure 1:**
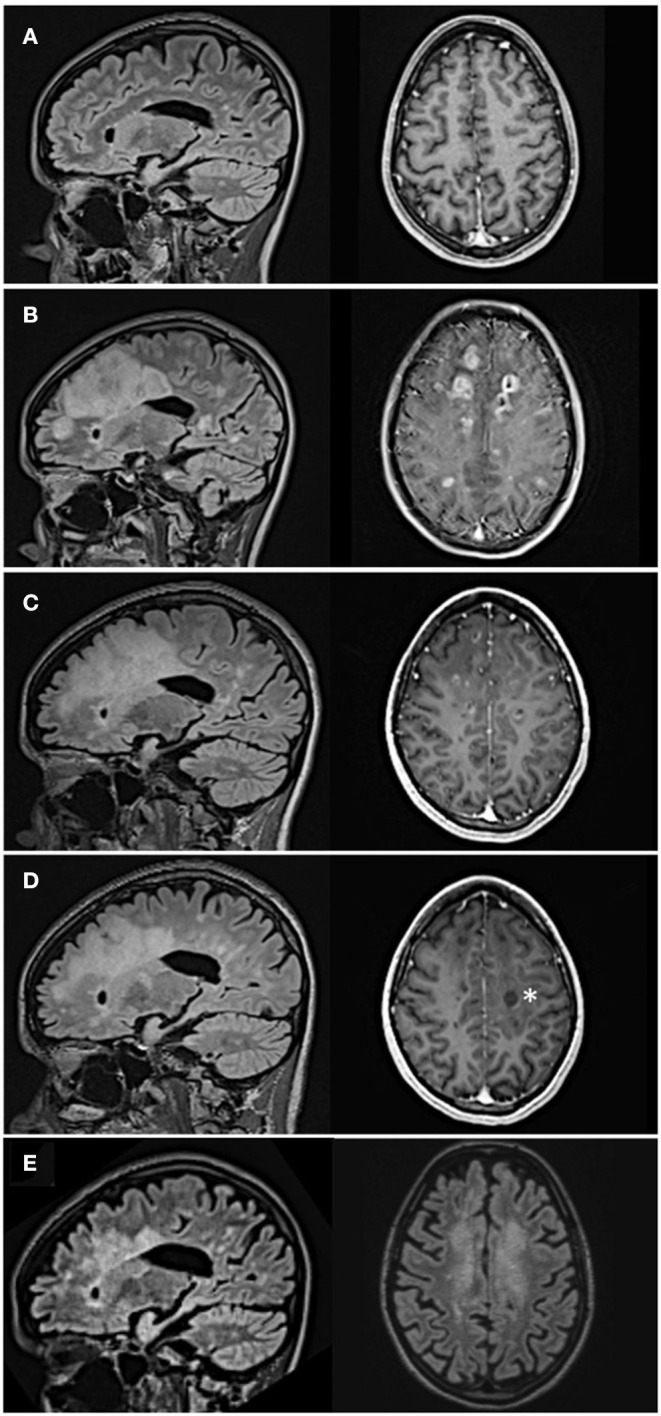
MR-imaging during disease course. Sagittal FLAIR- and axial T1 gadolinium sequences. **(A)** Moderate lesion load under fingolimod treatment. **(B)** Frontoparietal tumefactive lesions with disseminated Gadolinium-enhancement 6 weeks after discontinuation of fingolimod. **(C)** Remittent Gadolinium-enhancement after four cycles of plasmapheresis. **(D)** Further remission at re-admission 2 weeks after the last cycle of plasmapheresis; asterisk indicates active lesion. **(E)** Lesions decreasing in size 6 months after ocrelizumab initiation (only axial FLAIR available).

**Figure 2:**
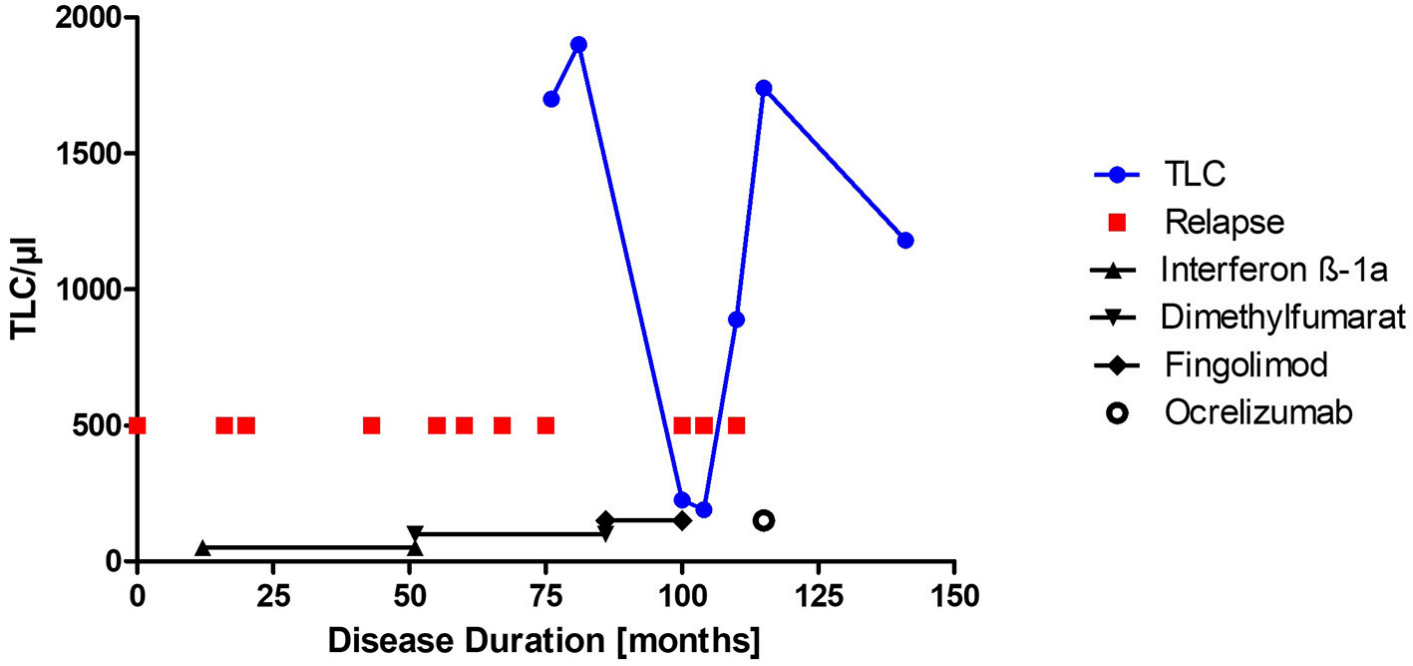
Clinical course and total lymphocyte count. Total lymphocyte count (TLC)/μl is depicted over the disease course. Relapses are shown as red squares. Duration of disease modifying therapies is shown with the black symbols, respectively. Ocrelizumab therapy was initiated and is still ongoing.

In a 26-year-old female patient with a 9-year history of RRMS, FTY treatment was discontinued due to lymphopenia of 225/μl and persisting disease activity in the form of optic neuritis. Prior to FTY she had been treated with interferon beta 1a and dimethylfumarate under which she had developed several relapses. Six weeks after FTY withdrawal the patient developed neuropsychiatric symptoms over the course of a few days including apathy, affective incontinence, and motoric aphasia. MRI revealed numerous tumefactive lesions with Gad enhancement and edema atypical of her prior disease course (Figure 1A prior MRI; Figure 1B MRI at admission). Under intravenous methylprednisolone therapy (1 g/d) over 5 days her neurological status deteriorated further. She could neither drink nor eat, suffered from psychomotor agitation and was unable to communicate with her caregivers, corresponding to an Expanded Disability Status Scale (EDSS) of 9.5. Her peripheral blood lymphocyte count at that time was 190/μl. After transfer to our clinic, we performed CSF analysis to rule out an infectious etiology. While we found a mild pleocytosis of 16/μl, three independent PCRs for JCV-DNA from serum and CSF were negative ruling out PML as a differential diagnosis. An extensive PCR and serological workup for borrelia, lues, and cryptococcosis was also negative, as well as anti-Aquaporin-4- and anti-MOG-antibodies. MR spectroscopy of a progressive lesion in the left frontal lobe yielded results suggestive of acute MS lesions (creatinin, cholin, and N-acetylaspartate slightly decreased, lactate slightly increased). The following day plasmapheresis was initiated, and the patient began to improve slowly. After the fourth plasmapheresis, she regained the ability to communicate and walk and was able to ingest small amounts of food. Follow-up MRI showed a decrease of the lesion size and number as well as the Gad enhancement (Figure 1C). After completion of seven plasmaphereses the patient could be transferred to a rehabilitation facility with an EDSS of 7.5. However, 2 weeks later she developed right sided hemiparesis. Corticosteroid therapy was started, and she was eventually readmitted to our hospital. Peripheral lymphocytes had increased to 890/μl but were still below the lower limit of normal. While MRI showed an overall decrease of Gad-enhancing lesions one subcortical lesion had remained active (Figure 1D, asterisk). As her symptoms had failed to improve under corticosteroids, a second cycle of seven plasmaphereses was initiated and the patient improved daily so that she was, again, released to a rehabilitation facility. The first dose of ocrelizumab (300 mg) was administered 7 weeks later. Six months after ocrelizumab initiation the patient remained relapse-free and showed clinical improvement now walking 1 km without help (EDSS 4.5). MRI showed a further remission with no remaining Gad enhancement (Figure 1E). Figure 2 shows an overview of the clinical course including relapses and DMT switches as well as total lymphocyte counts.

## Discussion

Several case reports describe tumefactive disease rebounds associated with fingolimod treatment (5) which may be linked to fingolimod-induced modulation of vascular permeability as observed in macular edema (6). However, the cases that occurred after FTY discontinuation are of particular interest as *post-hoc* analyses of the FREEDOMS and FREEDOMS II trials did not find a significantly increased risk of severe disease reactivation after FTY discontinuation (7). Sato et al. (8) presented a case series of 19 patients who were switched from fingolimod to dimethylfumarate. Ten of them experienced disease reactivation after cessation, with seven meeting the definition of rebound. Two patients suffered from persisting lymphopenia after 4 weeks but experienced no rebound. The seven rebound patients had a normal total lymphocyte count (TLC) at reactivation. However, most other case reports report either a normal lymphocyte count or provide no information on TLC. In rebound cases, it is hypothesized that rapid lymphocyte reconstitution and consecutive re-entry of lymphocytes into the CNS may cause an immune reconstitution inflammatory syndrome (IRIS)-like condition (9). This concept is corroborated by the fact that severe rebound events typically occur 2–4 months after therapy discontinuation at which time lymphocyte counts usually reach normal levels again. Furthermore, in mice FTY discontinuation can induce sphingosine-1-phosphate 1 (S1P1) overexpression in lymph-node entrapped lymphocytes leading to a massive sequential egress of lymphocytes (10). The resulting inflammatory spinal cord infiltrates are significantly higher than in vehicle-treated mice. However, in our patient disease reactivation occurred during persisting lymphopenia, which has been shown to be associated with low TCL before and under FTY treatment (11). In this context, the activity of brain-resident astrocytes may play a pivotal role. Giordana et al. (12) reported a fatal case of disease reactivation initially presenting with TDLs and symptoms similar to our patient. Autopsy revealed a strong S1P1 immunoreactivity on hypertrophic reactive astrocytes in active demyelinating lesions and in the periplaque normal appearing white matter (NAWM). The authors propose that upon FTY withdrawal, overexpression of S1P receptors on hyperactive astrocytes may lead to an activation of the pro-inflammatory transcription factor NFκB. A subsequent massive release of inflammatory cytokines and nitric oxide (NO) may explain the remarkable radiological characteristics and velocity of clinical deterioration. Due to an impaired blood brain barrier (BBB), these brain-derived inflammatory cytokines might diffuse into the peripheral blood where they would be cleared by plasmapheresis. This could explain the clinical improvement observed in our case. However, *in vitro* or *in vivo* data describing the precise mechanism of astrocyte cytokine release after FTY withdrawal is currently lacking. The main aspect to be learned from this case is that lymphocyte counts following FTY cessation are not a reliable tool to predict the probability of rebound. One of the most important risk factors is, however, persisting disease activity under FTY treatment flaring up after cessation (13). Moreover, with regard to impending disease reactivation a 6-week drug-free period before starting a new DMT could be too long even with persistent lymphopenia as stated by Bigaut et al. (14) in the Guidelines of the French Multiple Sclerosis Society. The authors recommend starting a new first-line therapy without a washout period and starting therapy with ocrelizumab and natalizumab after a washout period of 1 month. In both cases, TLC should not be taken into account concerning pre-therapeutic assessments. Thirdly, as suggested by Sato et al. (8), it is worthwhile to measure the ratio of TLCs even if that was not applicable in our case. This could help to decide when to start a new DMT after FTY cessation despite persisting lymphopenia. However, further studies are needed to define a cut-off value. Regarding differential diagnoses for tumefactive lesions the clinician should always rule out opportunistic infections such as herpes simplex virus (HSV), varicella zoster virus (VZV), cerebral cryptococcosis and PML. As of February 2020, 37 MS patients had contracted PML in the context of FTY therapy. Even though several studies could not confirm an increased infection rate due to lymphopenia (1) the European Medicines Agency (EMA) recommends discontinuation of FTY treatment when lymphocyte counts drop below 200/μl. Irrespective of these considerations, in the case presented here FTY discontinuation was indicated due to persisting disease activity.

## Conclusion

In summary, we conclude that more detailed and standardized guidelines are needed for either continuation or termination of therapy in persistent lymphopenia. However, a definitive potential risk factor for rebound to be considered is persistent disease activity under prior treatment (13). Secondly, the washout period after FTY cessation before starting a new DMT should not exceed 1 month and should not be dependent on TLC at the time of cessation (14). Finally, for the clinician it is important to bear in mind that neuropsychiatric symptoms in young MS patients might indicate disease rebound.

## Data Availability Statement

The raw data supporting the conclusions of this article will be made available by the authors, withoutundue reservation.

## Ethics Statement

Ethical review and approval was not required for the study on human participants in accordance with the local legislation and institutional requirements. The patients/participants provided their written informed consent to participate in this study. Written informed consent was obtained from the individual(s) for the publication of any potentially identifiable images or data included in this article.

## Author Contributions

VK, SM, and DK gave the idea of case reporting. VK and DK analyzed the case and prepared the MRI scans as well as the figure and the table. VK drafted the manuscript for intellectual content. MF, KB, EA, PA, OA, PK, SM, and DK critically reviewed the manuscript and were involved inpatients' healthcare. All the authors contributed to the article and approved the submitted version.

## Conflict of Interest

PA received compensation for serving on Scientific Advisory Boards for Allergan, Biogen, Celgene, Ipsen, Merck Serono, Merz Pharmaceuticals, Novartis, and Roche. He received speaker honoraria and travel support from Allergan, Bayer Vital GmbH, Biogen, Celgene, Ipsen, Merck Serono, Merz Pharmaceuticals, Novartis, Roche and research support from Allergan, Biogen, Celgene, Ipsen, Merck Serono, Merz Pharmaceuticals, Novartis, and Roche. OA has received grant support from Bayer, Biogen, Novartis, and Sanofi and consultancy or speaking fees and fees for serving on steering committees from Bayer, Biogen, Celgene, Medimmune, Merck, Novartis, Roche, Sanofi, and Teva. PK was supported by the Stifterverband/Novartisstiftung. SM received honoraria for lecturing and travel expenses for attending meetings from Almirall, Amicus Therapeutics Germany, Bayer Health Care, Biogen, Celgene, Diamed, Genzyme, MedDay Pharmaceuticals, Merck Serono, Novartis, Novo Nordisk, ONO Pharma, Roche, Sanofi-Aventis, Chugai Pharma, QuintilesIMS, and Teva. His research was funded by the German Ministry for Education and Research (BMBF), Deutsche Forschungsgemeinschaft (DFG), Else Kröner Fresenius Foundation, German Academic Exchange Service, Hertie Foundation, Interdisciplinary Center for Clinical Studies (IZKF) Muenster, German Foundation Neurology and by Almirall, Amicus Therapeutics Germany, Biogen, Diamed, Fresenius Medical Care, Genzyme, Merck Serono, Novartis, ONO Pharma, Roche, and Teva. DK received travel grants from GeNeuro and Merck, refund of congress participation fees from GeNeuro, Merck and Servier, consulting fees from Grifols, payment for lectures from Grifols, support for research projects from Teva and was funded by the Deutsche Forschungsgemeinschaft (DFG) while carrying research on human endogenous retroviruses at Cleveland Clinic. The remaining authors declare that the research was conducted in the absence of any commercial or financial relationships that could be construed as a potential conflict of interest.

## Publisher's Note

All claims expressed in this article are solely those of the authors and do not necessarily represent those of their affiliated organizations, or those of the publisher, the editors and the reviewers. Any product that may be evaluated in this article, or claim that may be made by its manufacturer, is not guaranteed or endorsed by the publisher.
